# Genetic Characterization in High-Risk Individuals from a Low-Resource City of Peru

**DOI:** 10.3390/cancers14225603

**Published:** 2022-11-15

**Authors:** Elizabeth Zavaleta, Nelly Solis, Maria Isabel Palacios, Liz Elva Zevallos-Escobar, Edison Vasquez Corales, Juan Carlos Bazo-Alvarez, Constantino Dominguez-Barrera, Anthony Campos, Patrik Wernhoff, Per Olaf Ekstrøm, Pål Møller, Tina Visnovska, Eivind Hovig, Janina Balazar-Palacios, Karin Alvarez-Valenzuela, Sigve Nakken, Mev Dominguez-Valentin

**Affiliations:** 1 Hospital La Caleta, Ministerio de Salud, Chimbote 02803, Peru; 2 Universidad Católica Los Ángeles de Chimbote, Chimbote 02801, Peru; 3 Research Department of Primary Care and Population Health, University College London, London WC1H 0NN, UK; 4 Escuela de Medicina, Universidad Cesar Vallejo, Piura 20001, Peru; 5 Independent Researcher, Lima 15468, Peru; 6 Department of Medical Genetics, Institute of Clinical Medicine, University of Oslo and Oslo University Hospital, 0450 Oslo, Norway; 7 Department of Tumor Biology, Institute for Cancer Research, Oslo University Hospital, 0450 Oslo, Norway; 8 Bioinformatics Core Facility, Oslo University Hospital, 0450 Oslo, Norway; 9 Centre for Bioinformatics, Department of Informatics, University of Oslo, 0450 Oslo, Norway; 10 Universidad Privada Norbert Wiener, Lima 15046, Peru; 11 Centro de Cáncer, Clínica Universidad de Los Andes, Santiago 7620157, Chile; 12 Centre for Cancer Cell Reprogramming, Institute of Clinical Medicine, Faculty of Medicine, University of Oslo, 0450 Oslo, Norway

**Keywords:** Peru, hereditary cancer, gene panel, family history

## Abstract

**Simple Summary:**

Genetic testing should be accessible to all individuals independently of where they live. There is an unbalanced distribution of resources and health care facilities in different geographic regions, not only when comparing high-income to low/middle-income countries but also within countries (e.g., rural vs. urban areas). Early age of onset is helpful for identifying patients who are affected by inherited syndromes and carry a pathogenic germline variant associated with cancer predisposition. Most of hereditary cancer mutations confer susceptibility to cancers in multiple organs. This study identified seven different hereditary cancer syndromes in a high-risk population located in a low-resource setting city and allow an appropriate genetic counselling and clinical management for these individuals and their relatives.

**Abstract:**

Background: Genetic testing for hereditary cancers is inconsistently applied within the healthcare systems in Latin America. In Peru, the prevalence and spectrum of cancer-predisposing germline variants is thus poorly characterized. Purpose: To determine the spectrum and prevalence of cancer-predisposing germline variants and variants of uncertain significance (VUS) in high-risk individuals located in a Peruvian low-resource setting city. Methods: Individuals presenting clinical criteria for hereditary cancer syndromes or being unaffected with familial history of cancer were included in the study. Samples from a total of 84 individuals were subjected to a high-throughput DNA sequencing assay that targeted a panel of 94 cancer predisposition genes. The pathogenicity of detected germline variants was classified according to the established American College of Medical Genetics and Genomics (ACMG) criteria. All pathogenic variants were validated by cycling temperature capillary electrophoresis. Results: We identified a total of eight pathogenic variants, found in 19 out of 84 individuals (23%). Pathogenic variants were identified in 24% (10/42) of unaffected individuals with family history of cancer and in 21% (9/42) of individuals with a cancer diagnosis. Pathogenic variants were identified in eight genes: *RET* (3), *BRCA1* (3), *SBDS* (2), *SBDS/MLH1* (4), *MLH1* (4), *TP53* (1), *FANCD2* (1), *DDB2/FANCG* (1). In cancer cases, all colon cancer cases were affected by pathogenic variants in *MLH1* and *SBDS* genes, while 20% (2/10) of the thyroid cancer cases by *RET* c.1900T>C variants were affected. One patient with endometrial cancer (1/3) had a double heterozygous pathogenic variant in *DDB2* and *FANCG* genes, while one breast cancer patient (1/14) had a pathogenic variant in *TP53* gene. Overall, each individual presented at least 17 VUS, totaling 1926 VUS for the full study population. Conclusion: We describe the first genetic characterization in a low-resource setting population where genetic testing is not yet implemented. We identified multiple pathogenic germline variants in clinically actionable predisposition genes, that have an impact on providing an appropriate genetic counselling and clinical management for individuals and their relatives who carry these variants. We also reported a high number of VUS, which may indicate variants specific for this population and may require a determination of their clinical significance.

## 1. Introduction

Clinical genetic testing for cancer-risk assessment has become widespread over the last two decades, with evidence–based testing guidelines for hereditary breast cancer (BC), Lynch syndrome (LS), Li-Fraumeni syndrome (LF) syndrome, familial adenomatous polyposis, hereditary diffuse gastric cancer, and a few other conditions [1].

Often, an early age of onset is helpful for identifying patients who are affected by inherited syndromes and that carry cancer-predisposing germline variants. It is also known that most hereditary cancer mutations confer susceptibility to cancer in multiple organs [1]. Some ethnic groups have been described to be at greater risk of developing cancers, such as individuals of Ashkenazi Jewish descent being at increased risk of developing early-onset breast and ovarian cancer [2,3].

In a rural and low-income population from Northern Peru, we have previously identified a greater proportion of cancer having a young age of onset and having a differential profile of the most frequent cancers (e.g., submaxillary gland, stomach cancer, endometrial cancer) [4]. Therefore, there is a need to identify patients who are affected by inherited syndromes and carry a cancer-predisposing germline variant. However, genetic testing is not widely available at the health-care system in Peru and no study has until now assessed the prevalence and mutational spectrum of germline variants in high-risk individuals from rural and low-income populations.

Targeted multigene next generation sequencing (NGS) panels have a significant impact on the accessibility of genetic testing owing to their versatility and low cost [5]. Given the immediate clinical management implications that patients with genetic disorders may have [5], these data are urgently needed to inform genetic counseling. To that end, a relatively large cancer panel of 94 cancer predisposition genes was sequenced in a population of 84 high-risk Peruvian individuals. In addition, sociodemographic data have been collected from the study population.

## 2. Methods

### 2.1. Study Population

The study population (n = 101) was selected from three regional hospitals that cares for the rural and low-income population from Northern Peru (Chimbote).

The selection criteria for the 101 individuals were as follows:

- Having an early onset of cancer (cancer diagnosis < 55 years of age) (n = 51)
- Having a late onset of cancer and familial history of cancer (n = 3)
- Being unaffected individuals with familial history of cancer (n = 47)

Ethical approval for the study was granted by the Institutional Committee on Research Ethics from Universidad Católica Los Angeles de Chimbote (ref 051-2018) and by the Norwegian Regional Committee for Medical and Health Research Ethics (ref 185588). All examined patients had signed an informed consent for their participation in the study.

The participants completed a survey that contained questions about sociodemographic characteristics, family history, lifestyle habits and social determinants of health (quality of housing and health care assurance).

### 2.2. Next Generation Sequencing (NGS)

Genomic DNA was isolated from peripheral blood samples using DNeasy Blood & Tissue Kit (Qiagen, Germantown, MD, USA), according to the manufacturer’s protocol. Whole genome sequencing libraries were made by the Oslo University Hospital Genomics Core Facility (oslo.genomics.no, accessed on 12 October 2022) using Illumina Nextera DNA Flex Pre-Enrichment Library Prep kit and captured using the Illumina TruSight Cancer panel, which enriches for a total of 94 protein-coding genes known to be implicated with cancer risk. One hundred nanograms of genomics DNA was used as input material and libraries were prepared following manufacturer’s instructions. The resulting libraries were sequenced paired-end 2 × 149 bp on a NextSeq 500 using a Mid Output Kit v2.5 kit from Illumina (San Diego, CA, USA).

### 2.3. Variant Detection and Interpretation

Small germline variants (single nucleotide variants (SNVs)/short insertions and deletions (indels)) were identified with the DRAGEN BIO-IT platform from Illumina (software version 01.011.565.3.6.3). We executed the DNA analysis pipeline, including quality checks of the raw sequencing data, read mapping towards the GRCh37 reference genome and variant calling. The targeted regions of the Illumina TruSight Cancer panel were extended by 250 bp on both ends, and otherwise running the pipeline with the default settings. Several quality filters were automatically applied on the called variants, and the variants not passing the filters were marked as such. All the variants with low sequencing depth (less than 2) were marked. Additionally, the SNVs with variant quality below 10.41 and indels with variant quality below 7.83 were also marked. During the data analysis, multiple quality metrics were reported allowing us to inspect the quality of the sequenced data, subsequently provided in Section 3.

We used the Cancer Predisposition Sequencing Reporter (CPSR) v0.6.1 for variant interpretation [6]. In brief, CPSR classifies the clinical significance of variants according to a standard five-tier scheme, i.e., benign (B), Likely Benign (LB), variants of uncertain significance (VUS), likely pathogenic (LP), and pathogenic (P). For variants that are found with existing submissions in ClinVar, CPSR assigns the consensus classification reported in ClinVar. For novel variants (i.e., not present in ClinVar), CPSR assigns a classification according to a comprehensive implementation of American College of Medical Genetics (ACMG) guidelines for variant interpretation [7]. Considering that the DNA samples originated from Peruvian families, we used the admixed American (AMR) sub-population of gnomAD as the reference source of variant population frequencies.

### 2.4. Variant Validation by Cycling Temperature Capillary Electrophoresis (CTCE)

The pathogenic variants identified in this study were validated by cycling temperature capillary electrophoresis (CTCE) or real-time PCR amplification by allele specific PCR. CTCE is based on allele separation by cooperative melting equilibrium while cycling the temperature around the melting temperature using capillary technology [8]. The heterozygote samples display two peaks when separated by CTCE. This approach has previously been described and extensively used to detect somatic mutations and single nucleotide polymorphisms (SNPs) [9,10,11]. The amplicon design was performed by the variant melting profile tool (https://hyperbrowser.uio.no/hb/?toolid=hb_variant_melting_profiles/, accessed on 12 October 2022) [10]. A variant not suited for CTCE analysis was alternatively verified using allele-specific real-time PCR. The samples where assayed in two different reactions with primers specific for each allele interrogated. Cycle threshold (Ct) below 25 was used as cut off for scoring of amplification of the specific alleles. Blank and template not having the variant was used as controls. Primer sequences, PCR reaction conditions and electrophoresis settings are available upon request.

## 3. Results

### 3.1. Sociodemographic Characteristics of the Study Population

We examined a total of 101 individuals, mostly women (83.2%, n = 84) and younger than 50 years of age (78.2%, n = 79). The population was essentially non-white (92.5%, n = 86), living in an urban area (91.4%, n = 85) with 7 or more years of formal education (94.6%, n = 88). Only a few persons had private health insurance (2.2%, n = 2) or oncologic insurance (6.5%, n = 6), although 15.1% (n = 14) reported having one or more chronic diseases. Other characteristics are detailed in Table 1.

When analyzed for the presence of any cancer in the study population (n = 101), people > 50 years of age (86.4%, 19/22), women (58.3%, 49/84), white (85.7%, 6/7), and with a higher family income (64.1%, n = 25/39) presented a higher prevalence of cancer diagnosis than their respective counterparts. Own tenancy house (60.0%, 36/60) and nuclear family type (63.0%, 29/46) characteristics were mostly observed in individuals with a diagnosis of cancer. People having chronic diseases (85.7%, 12/14) or being overweight/obese (73.5%, 25/34) were found to have a higher prevalence of cancer diagnosis (Table 1).

### 3.2. Clinical Characteristics of the Individuals Tested by Gene Panel

**Figure 1:**
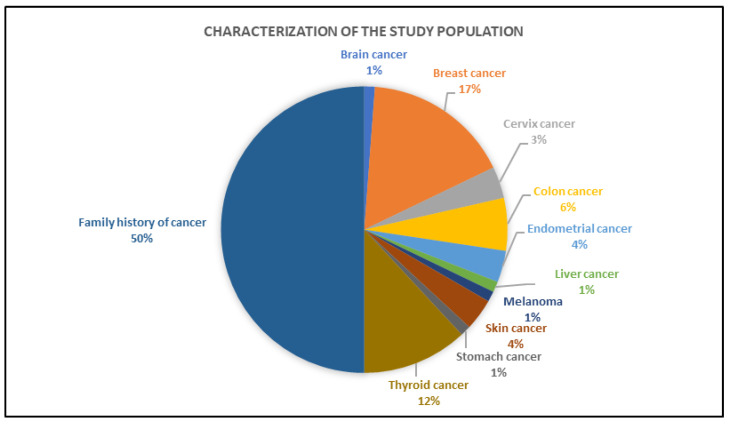
Clinical characterization of the study population.

Out of the 101 individuals, 84 were analyzed by gene panel testing, while 17 cases were excluded due to poor quality of DNA for NGS. The most common cancer sites of origin were breast (33%, 14/42), followed by thyroid (24%, 10/42) and colon (12%, 5/42) (Figure 1). The median age at first cancer diagnosis was 35 years (range 4–69 years). Half of the cases (42/84) were unaffected individuals with a familial history of cancer. Females (86%, 72/84) were more commonly affected than males (14%, 12/84).

### 3.3. NGS Data Analysis

The gene panel sequencing generated on average 4.4 million reads per sample. The proportion of the marked duplicates ranged from 14% to 29% of the total sample reads, and the proportion of the reads mapping to the reference genome with MAPQ at least 40 ranged from 93% to almost 97%. The mean depth of coverage of the targeted regions ranged from 169× to 455×, while the percentage of the targeted regions with depth of at least 25× ranged from 89% to 93% between individuals. On average, 1344 variants were called per sample. For a more comprehensive summary of the metrics related to the primary analysis of the sequencing data, see Supplementary Table S1.

### 3.4. Pathogenic Germline Findings

We identified a total of eight pathogenic variants, found in 19 out of 84 individuals (23% of all samples subject to sequencing) belonging to ten different families. Pathogenic variants were identified in 24% (10/42) of unaffected individuals with a family history of cancer and in 21% (9/42) of individuals with a cancer diagnosis.

**Figure 2:**
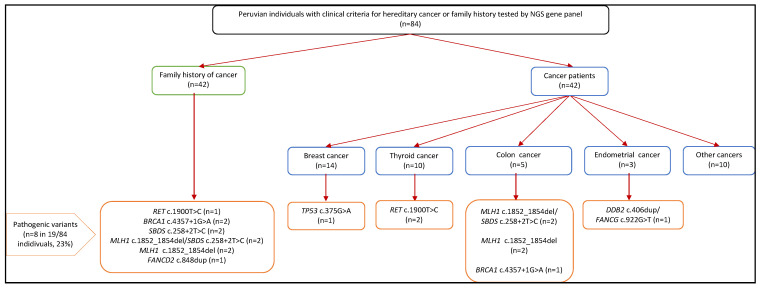
Flowchart of the study population submitted to NGS and results from the study.

The pathogenicity for most of these variants was previously reported by ClinVar and in this study, we report *DDB2* (ENST00000256996.4: c.406dup), *FANCG* (ENST00000378643.3: c.922G>T) and *FANCD2* (ENST00000287647.3: c.848dup) as novel pathogenic variants according to the CPSR’s classification (Table 2). The genes affected by pathogenic variants included *RET* (n = 3 individuals), *BRCA1* (n = 3), *SBDS* (n = 6), *MLH1* (n = 8), *DDB2* (n = 1), *FANCG* (n = 1), *FANCD2* (n = 1) and *TP53* (n = 1), see also Table 2 and Figure 2.

**Figure 3:**
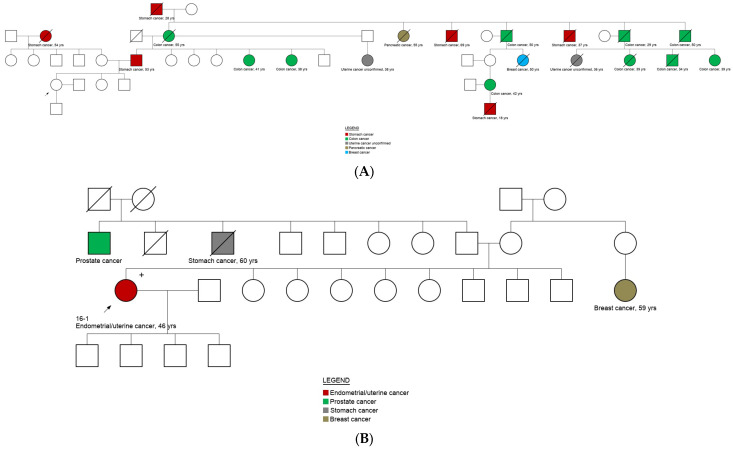
Pedigrees of carriers of double pathogenic heterozygous variants. (**A**) Family pedigree where the proband carried the variants *MLH1* c.1852_1854del and *SBDS* c.258+2T>C (ID: 27-4). +: Carrier of *MLH1* (ENST00000231790.2) c.1852_1854del and/or *SBDS* (ENST00000246868.2) c.258+2T>C variants (detailed description in Table 2). (**B**) Family pedigree where the proband carried the variants *DDB2* c.406dup and *FANCG* c.922G>T (ID: 16-1). +: Carrier of the variants *DDB2* c.406dup and *FANCG* c.922G>T.

All pathogenic variants were confirmed by CTCE, showing 100% concordance. A total of five individuals carried double heterozygous pathogenic variants, e.g., *MLH1* and *SBDS* variants were found in three related family members and in one unrelated individual. *DDB2* and *FANCG* were identified in a patient with a diagnosis of endometrium cancer at 45 years (see pedigrees in Figure 3).

The NGS results revealed that each individual carried an average of 23 VUS (range 17–34) in the set of 94 cancer susceptibility genes.

### 3.5. Genotype and Phenotype Correlation

The variant in the *TP53* (ENST00000269305.4: c.375G>A) gene is a silent type of variant that, based on its spliceogenic position, has been reported as pathogenic [12]. It was identified in a patient with a breast cancer diagnosis and is associated to the LF syndrome phenotype. A double heterozygous frameshift and missense pathogenic variant in *DDB2* and *FANCG* genes, respectively were identified in a patient with endometrial cancer, which contrasts to the reported associated phenotype for these variants (Xeroderma pigmentosum and Fanconi anemia, respectively). The *RET* pathogenic variant (ENST00000355710.3) c.1900T>C) was identified in three related cases with a family history of thyroid cancer, providing a diagnosis of multiple endocrine neoplasia type 2 (MEN2), which is characterized by the development of medullary thyroid carcinoma [13]. Interestingly, the splice site variant in *BRCA1* gene (ENST00000471181.2: c.4357+1G>A) was identified in three related cases, all had a family history of colon, stomach, thyroid and pancreatic cancer without the presence of breast or ovarian cancer in the family (Supplementary Figure S1). A pathogenic frameshift deletion in *MLH1* (ENST00000231790.2: c.1852_1854del) was identified in 8 individuals, providing a LS diagnosis. In addition, half of these cases (4/8) also carried the *SBDS* (ENST00000246868.2: c.258+2T>C) variant, while two cases without a cancer diagnosis only carried the heterozygous *SBDS* c.258+2T>C variant. Biallelic pathogenic variants in *SBDS* have been associated to the Schwachman-Diamond syndrome 1 (SBDS) that has a variety of clinical features, including exocrine pancreatic insufficiency and hematological dysfunction [14,15]. The *FANCD2* (ENST00000287647.3: c.848dup) pathogenic variant was identified in a case with family history of breast, liver lung and colon cancer and has been associated to Fanconi anemia (Table 2).

## 4. Discussion

This study has for the first time allowed the identification of seven different hereditary cancer syndromes in a high-risk population located in a low-resource setting city where genetic testing is not available. Importantly, we could provide information as to which genes may have been causative for cancer in the patients and their relatives. This is likely to have direct impact on providing an appropriate genetic counselling and clinical management for individuals and their relatives carrying these variants, depending on available counselling.

We are aware that social and economic factors have a greater influence on health than clinical care [16,17]. In this study, we described that public health assurance was reported to be granted for most of the individuals (90%), while private oncological assurance was obtained only for 6.5%. According to the Northern Peru Cancer Registry (IREN Norte), 22,250 cases of neoplasms have been described in the period of 2007–2021. Approximately 6% of these cases come from Chimbote cancer hospitals [18]. Chimbote is the largest city and port of Ancash (department and region in Northern Peru) and has an estimated population (2015) of 371,012 inhabitants [19]. Interestingly, half of the reported cancer cases from IREN Norte have been diagnosed in people aged up to 59 years. In Chimbote, patients with a suspected diagnosis of cancer need to travel to the larger cities such as Lima (capital, 428 km/266 miles) or Trujillo (132 km/82 miles) to have an accurate cancer diagnosis and initiate their treatments in larger public hospitals. In this study, patients have reported that the transference to a cancer-based hospital takes up to 3 years. These social needs contribute to health inequities and higher health care costs [16,17]. Our results indicate a need for urgent implementation of genetic testing and counselling in public hospitals/centres located in low-resource setting cities to provide an early diagnosis and personalized treatment to cancer patients. 

The lack of the genetic knowledge prevents effective prevention for hereditary cancer syndromes. In this study, we have provided the genetic information for 19 individuals/families that will benefit from personalized cancer medicine. We demonstrated a rate of pathogenic variants (23%) within the reported range from other populations [20,21,22]. Interestingly, a high number of VUS was identified in this study that suggest a need for their clinical significance classification. There is a need for more genetic information from delineated populations that have not been previously characterized, it will allow interpretation of genetics findings and their cancer-associations in order to provide a properly genetic counselling.

Individuals with the *RET* c.1900T>C (p.Cys634Arg) should follow the recommendations by the American Thyroid Association (ATA) regarding surveillance and management. These include screening, surgery, therapy and also consideration of the implications for family members regarding reproductive considerations [23]. Carriers of pathogenic variants in *BRCA1* have a high risk (approximately > 60%) of developing breast cancer, followed by ovarian cancer (39–58%), and around 5% develop pancreatic cancer. Management and surveillance should be undertaken according to the National Comprehensive Cancer Network (NCCN). The management guidelines for LS have recently been updated and are based on gene and gender-specific risks, with a resultant good prognosis for the most commonly associated cancers [24,25]. In our cohort, we identified four LS carriers with the *SBDS* c.258+2T>C variant, which has not previously been associated to LS, but could affect the risk for these patients. There is a need to understand the association of *SBDS* and *MLH1* in LS in order to facilitate personalized medicine for the carriers. On the other hand, *SBDS* pathogenic variants have been associated to the autosomal recessive syndrome Shwachman-Diamond 1, that is an inherited bone marrow failure syndrome characterized by enhanced cancer predisposition [26]. LF is generally caused by pathogenic germline variants in *TP53*, which are identified in ~70% of families meeting the classic LF diagnostic criteria [27] and well-established clinical and surveillance recommendations exist.

Pathogenic variants in *FANCG* and *FANCD2* genes have a pathogenic variant frequency of 8% and 4%, respectively, and have shown to exert an autosomal recessive effect [28]. Monoallelic pathogenic variants in *FANCD1/BRCA2*, *FANCS/BRCA1*, *FANCJ/BRIP1*, *FANCM*, *FANCN/PALB2*, and *FANCO/RAD51C* have been linked to familial breast and ovarian cancer [29]. DDB2 (damage-specific DNA-binding protein 2, also known as the p48 subunit) is ubiquitously present in human tissues, albeit differentially expressed [30] and has been recently associated to many cancers, including prostate, colorectal, skin, ovarian, head and neck, suggesting a critical role for DDB2 in tumor suppression [30]. There is a need to further understand the involvement of *DDB2*, *FANCG* and *FANCD2* with respect to cancer risk, in order to facilitate personalized medicine for the carriers.

## 5. Conclusions

Improving the understanding of the genetics of inherited cancer in low income countries such as Peru is crucial to harvest a significant number of individuals with pathogenic variants in clinically actionable genes. The results obtained in this study had a significant impact on patients and their relatives since it allowed genetic counselling and personalized management decisions.

## Figures and Tables

**Table 1:** Characteristics of the study population.

Characteristics		Overall (n = 101)		No Cancer Diagnosis (n = 47)		Cancer Diagnosis (n = 54)	
		n	%	n	%	n	%
**Sociodemographic characteristics**							
Age							
	<50 years	79	78.2	44	55.7	35	44.3
	>50 years	22	21.8	3	13.6	19	86.4
Gender							
	Women	84	83.2	35	41.7	49	58.3
	Men	17	16.8	12	70.6	5	29.4
Ethnicity *							
	White	7	7.5	1	14.3	6	85.7
	Non-white	86	92.5	38	44.2	48	55.8
Educational level *							
	<7 years of education	5	5.4	0	0.0	5	100.0
	≥7 years of education	88	94.6	39	44.3	49	55.7
Professional level **							
	College/University Student	11	12.1	10	90.9	1	9.1
	Undergraduate (finished)	25	27.5	11	44.0	14	56.0
	Technical (finished)	12	13.2	5	41.7	7	58.3
	Primary/secondary education	43	47.3	11	25.6	32	74.4
Work condition *							
	Hired by a company	19	20.4	9	47.4	10	52.6
	Independent worker (freelance)	8	8.6	2	25.0	6	75.0
	Independent worker (entrepreneur)	15	16.1	6	40.0	9	60.0
	Domestic worker	2	2.2	0	0.0	2	100.0
	Housewife	44	47.3	21	47.7	23	52.3
	Not able to work	5	5.4	1	20.0	4	80.0
Family income (monthly) *							
	High	39	41.9	14	35.9	25	64.1
	Low	54	58.1	25	46.3	29	53.7
Family income source *							
	Head of home (1 person)	52	55.9	22	42.3	30	57.7
	Spouses (2 persons)	28	30.1	12	42.9	16	57.1
	Mixed (3 or more persons)	13	14.0	5	38.5	8	61.5
House location *							
	Urban area	85	91.4	35	41.2	50	58.8
	Marginal-urban area	1	1.1	0	0.0	1	100.0
	Rural area	7	7.5	4	57.1	3	42.9
Type of house *							
	For one family only	42	45.2	15	35.7	27	64.3
	For two or more families	51	54.8	24	47.1	27	52.9
House tenancy *							
	Own house	60	64.5	24	40.0	36	60.0
	Rented house	15	16.1	7	46.7	8	53.3
	Occupied house (no payment)	18	19.4	8	44.4	10	55.6
Type of family *							
	Nuclear (couple and children)	46	49.5	17	37.0	29	63.0
	Extended (more than one nuclear family)	47	50.5	22	46.8	25	53.2
Number of persons per house *							
	Less than 3 persons	5	5.4	2	40.0	3	60.0
	3 to 5 persons	55	59.1	19	34.5	36	65.5
	6 or more persons	33	35.5	18	54.5	15	45.5
**Health services and insurance**							
Health services							
	ESSALUD III	24	23.8	11	45.8	13	54.2
	Hospital La Caleta	73	72.3	35	47.9	38	52.1
	Hospital Regional	4	4.0	1	25.0	3	75.0
Health insurance *							
	Seguro Integral de Salud (SIS)	57	61.3	22	38.6	35	61.4
	Seguro Social de Salud (EsSalud)	27	29.0	11	40.7	16	59.3
	Private Insurance	2	2.2	0	0.0	2	100.0
	None	7	7.5	6	85.7	1	14.3
Oncologic insurance *							
	Yes	6	6.5	5	83.3	1	16.7
	No	87	93.5	34	39.1	53	60.9
**Health and lifestyle**							
Chronic disease(s) *							
	Yes	14	15.1	2	14.3	12	85.7
	No	79	84.9	37	46.8	42	53.2
Weight *							
	Normal	59	63.4	30	50.8	29	49.2
	Overweight or obesity	34	36.6	9	26.5	25	73.5

(*) 8 missing values. (**) 10 missing values. Family income (monthly): High was defined above 400 USD.

**Table 2:** Description of the 8 pathogenic variants found in 19 high-risk individuals analyzed by a panel of 94 cancer predisposition genes cancer.

Selection Criteria for the Study	Age at Cancer Diagnosis	Grade	Gene Variant	Protein Change	ACMG Classification	ACMG Evidence Code *	Pathogenicity Reported by	Reported Associated Phenotype	Inheritance Pattern
Breast	34	III	*TP53* (ENST00000269305.4) c.375G>A	p.Thr125 =	Pathogenic	PM2_2, PP3	Clinvar	Hereditary cancer-predisposing syndrome; Rhabdomyosarcoma (disease); Li-Fraumeni syndrome	Autosomal dominant
Endometrial	45	na	*DDB2* (ENST00000256996.4) c.406dup	p.Ile136AsnfsTer30	Pathogenic	PM2_2, PVS1_1	CSPR	Xeroderma pigmentosum, group E, DDB-negative subtype	Autosomal recessive
			*FANCG* (ENST00000378643.3) c.922G>T	p.Glu308Ter	Pathogenic	PM2_2, PVS1_1	CSPR	Fanconi anemia, complementation group G	
Thyroid	22	na	*RET* (ENST00000355710.3) c.1900T>C	p.Cys634Arg	Pathogenic	PM2_1, PS1, PP3	Clinvar	Multiple endocrine neoplasia type 2	Autosomal dominant
Familial history of cancer	na		*RET* (ENST00000355710.3) c.1900T>C	p.Cys634Arg	Pathogenic	PM2_1, PS1, PP3	Clinvar	Multiple endocrine neoplasia type 2	Autosomal dominant
Thyroid	18	na	*RET* (ENST00000355710.3) c.1900T>C	p.Cys634Arg	Pathogenic	PM2_1, PS1, PP3	Clinvar	Multiple endocrine neoplasia type 2	Autosomal dominant
Colon	51	III	*BRCA1* (ENST00000471181.2) c.4357+1G>A		Pathogenic	PM2_1, PVS1_7, PP3	Clinvar	Hereditary cancer-predisposing syndrome; Hereditary breast and ovarian cancer syndrome; Breast-ovarian cancer, familial 1	Autosomal dominant
Familial history of cancer	na		*BRCA1* (ENST00000471181.2) c.4357+1G>A		Pathogenic	PM2_1, PVS1_7, PP3	Clinvar	Hereditary cancer-predisposing syndrome; Hereditary breast and ovarian cancer syndrome; Breast-ovarian cancer, familial 1	Autosomal dominant
Familial history of cancer	na		*BRCA1* (ENST00000471181.2) c.4357+1G>A		Pathogenic	PM2_1, PVS1_7, PP3	Clinvar	Hereditary cancer-predisposing syndrome; Hereditary breast and ovarian cancer syndrome; Breast-ovarian cancer, familial 1	Autosomal dominant
Familial history of cancer	na		*SBDS* (ENST00000246868.2) c.258+2T>C		Pathogenic	BS1, PP3	Clinvar	Shwachman-Diamond syndrome 1	Autosomal recessive
Familial history of cancer	na		*MLH1* (ENST00000231790.2) c.1852_1854del	p.Lys618del	Pathogenic	PM2_1, PS1, PM4	Clinvar	Lynch syndrome	Autosomal dominant
			*SBDS* (ENST00000246868.2) c.258+2T>C		Pathogenic	BS1, PP3	Clinvar	Shwachman-Diamond syndrome 1	Autosomal recessive
Colon	41	II	*MLH1* (ENST00000231790.2) c.1852_1854del	p.Lys618del	Pathogenic	PM2_1, PS1, PM4	Clinvar	Lynch syndrome	Autosomal dominant
			*SBDS* (ENST00000246868.2) c.258+2T>C		Pathogenic	BS1, PP3	Clinvar	Shwachman-Diamond syndrome 1	Autosomal recessive
Familial history of cancer	na		*MLH1* (ENST00000231790.2) c.1852_1854del	p.Lys618del	Pathogenic	PM2_1, PS1, PM4	Clinvar	Lynch syndrome	Autosomal dominant
			*SBDS* (ENST00000246868.2) c.258+2T>C		Pathogenic	BS1, PP3	Clinvar	Shwachman-Diamond syndrome 1	Autosomal recessive
Familial history of cancer	na		*SBDS* (ENST00000246868.2) c.258+2T>C		Pathogenic	BS1, PP3	Clinvar	Shwachman-Diamond syndrome 1	Autosomal recessive
Colon	38	I	*MLH1* (ENST00000231790.2) c.1852_1854del	p.Lys618del	Pathogenic	PM2_1, PS1, PM4	Clinvar	Lynch syndrome	Autosomal dominant
			*SBDS* (ENST00000246868.2) c.258+2T>C		Pathogenic	BS1, PP3	Clinvar	Shwachman-Diamond syndrome 1	Autosomal recessive
Colon	42	I	*MLH1* (ENST00000231790.2) c.1852_1854del	p.Lys618del	Pathogenic	PM2_1, PS1, PM4	Clinvar	Lynch syndrome	Autosomal dominant
Colon	39	II	*MLH1* (ENST00000231790.2) c.1852_1854del	p.Lys618del	Pathogenic	PM2_1, PS1, PM4	Clinvar	Lynch syndrome	Autosomal dominant
Familial history of cancer	na		*MLH1* (ENST00000231790.2) c.1852_1854del	p.Lys618del	Pathogenic	PM2_1, PS1, PM4	Clinvar	Lynch syndrome	Autosomal dominant
Familial history of cancer	na		*MLH1* (ENST00000231790.2) c.1852_1854del	p.Lys618del	Pathogenic	PM2_1, PS1, PM4	Clinvar	Lynch syndrome	Autosomal dominant
Familial history of cancer	na		*FANCD2* (ENST00000287647.3)c.848dup	p.Phe284ValfsTer13	Pathogenic	PM2_2, PVS1_1	CSPR	Fanconi anemia, complementation group D2	Autosomal recessive

na: not available; DDB2: DNA damage-binding protein 2; CPSR: Cancer Predisposition Sequencing Reporter. * ACMG evidence codes attributed to each variant according the implementation in CPSR https://sigven.github.io/cpsr/articles/variant_classification.html, accessed on 12 October 2022.

## Data Availability

Not applicable.

## Supplementary Materials

The following supporting information can be downloaded at: https://www.mdpi.com/article/10.3390/cancers14225603/s1, Figure S1: Family pedigree where the proband carried the variant *BRCA1* (ENST00000471181.2) c.4357+1G>A; Table S1: Summary of various quality metrics related to the primary analysis of the 84 analysed NGS samples
