# Supplementary material for: Genetic Characterization in High-Risk Individuals from a Low-Resource City of Peru
Source: Cancers (Basel). 2022 Nov 15;14(22):5603. doi: 10.3390/cancers14225603 (PMC9688598; doi:10.3390/cancers14225603)
Supplement: Supplementary file 1 [file cancers-14-05603-s001.zip › cancers-1995009-supplementary.pdf]

cancers

MDPI

Supplementary Material: Genetic Characterization in High-Risk Individuals from a Low-Resource City of Peru

Elizabeth Zavaleta, Nelly Solis, Maria Isabel Palacios, Liz Elva Zevallos-Escobar, Edison Vasquez Corales, Juan Carlos Bazo-Alvarez, Constantino Dominguez-Barrera, Anthony Campos, Patrik Wernhoff, Per Olaf Ekstrøm, Pål Møller, Tina Visnovska, Eivind Hovig, Janina Balazar-Palacios, Karin Alvarez-Valenzuela, Sigve Nakken and Mev Dominguez-Valentin

Supplementary figure S1. Family pedigree where the proband carried the variant *BRCA1* (ENST00000471181.2) c.4357+1G>A.

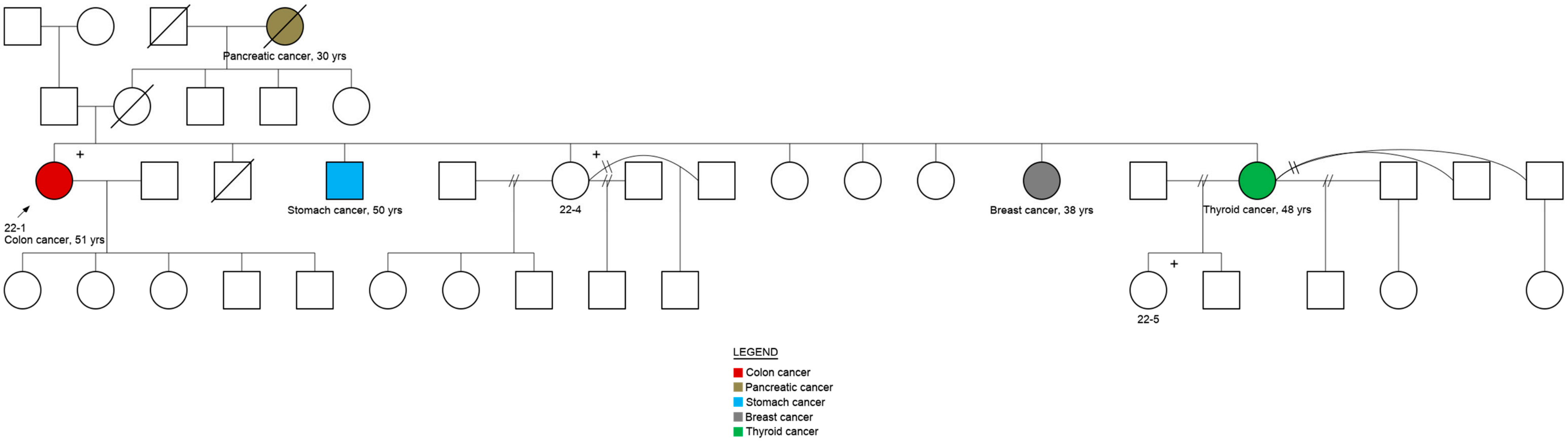

+: Carrier of *BRCA1* (ENST00000471181.2) c.4357+1G>A variant.

Supplementary Table S1. Summary of various quality metrics related to the primary analysis of the 84 analysed NGS samples.

SampleID	TotalReadCount	MarkedDuplicatesCount	MarkedDuplicates (%)	UniqueMappedCount	UniqueMapped (%)	MAPQ>39Count	MAPQ>39%	TargetMeanCoverage	SmallVariantsCount
**SAMPLE_01**	4306702	808172	18.77	3494143	81.13	4072617	94.56	329.26	1352
**SAMPLE_02**	5422346	1218150	22.47	4198994	77.44	5121517	94.45	407.48	1435
**SAMPLE_03**	4136992	780680	18.87	3351539	81.01	3888460	93.99	324.00	1287
**SAMPLE_04**	4217184	819023	19.42	3395240	80.51	3988817	94.58	330.65	1434
**SAMPLE_05**	3154060	510588	16.19	2641272	83.74	2974170	94.30	256.87	1426
**SAMPLE_06**	3667028	666226	18.17	2996904	81.73	3484546	95.02	296.26	1425
**SAMPLE_07**	4286010	832490	19.42	3449507	80.48	4041816	94.30	335.17	1338
**SAMPLE_08**	4400140	850979	19.34	3544581	80.56	4172516	94.83	344.74	1419
**SAMPLE_09**	3858364	707594	18.34	3146318	81.55	3638934	94.31	306.46	1356
**SAMPLE_10**	3988152	761105	19.08	3223256	80.82	3762974	94.35	310.24	1339
**SAMPLE_11**	4537970	946067	20.85	3587637	79.06	4288345	94.50	347.24	1294
**SAMPLE_12**	5362140	1244115	23.20	4112643	76.70	5084934	94.83	398.73	1412
**SAMPLE_13**	5410000	1355376	25.05	4048529	74.83	5228801	96.65	406.85	1369
**SAMPLE_14**	4472046	946908	21.17	3520519	78.72	4253573	95.11	345.48	1449
**SAMPLE_15**	4752614	1035745	21.79	3712154	78.11	4502667	94.74	362.38	1407
**SAMPLE_16**	3537878	629622	17.80	2904750	82.10	3321152	93.87	278.68	1295
**SAMPLE_17**	4839334	1073494	22.18	3760264	77.70	4572859	94.49	364.52	1405
**SAMPLE_18**	4707644	1166239	24.77	3536926	75.13	4452290	94.58	335.19	1212
**SAMPLE_19**	4363940	991754	22.73	3366396	77.14	4210221	96.48	343.78	1410
**SAMPLE_20**	4392822	885992	20.17	3502380	79.73	4155814	94.60	339.47	1358
**SAMPLE_21**	4147762	900059	21.70	3241837	78.16	4015566	96.81	338.09	1267
**SAMPLE_22**	4825444	1060434	21.98	3760443	77.93	4585233	95.02	366.01	1282
**SAMPLE_23**	5860556	1671934	28.53	4183130	71.38	5494262	93.75	395.39	1197
**SAMPLE_24**	5067370	1255720	24.78	3806225	75.11	4901888	96.73	393.60	1388
**SAMPLE_25**	3363226	670952	19.95	2688266	79.93	3254142	96.76	290.19	1396
**SAMPLE_26**	3458826	613676	17.74	2841369	82.15	3335661	96.44	271.70	1338
**SAMPLE_27**	3695812	674941	18.26	3018316	81.67	3482143	94.22	291.02	1344
**SAMPLE_28**	4989922	1280004	25.65	3704665	74.24	4817971	96.55	384.11	1368
**SAMPLE_29**	4485526	947248	21.12	3534256	78.79	4264763	95.08	344.69	1424
**SAMPLE_30**	5039630	1152117	22.86	3882771	77.04	4785938	94.97	377.40	1518
**SAMPLE_31**	4235578	821213	19.39	3410728	80.53	3987354	94.14	323.62	1283
**SAMPLE_32**	4177210	845637	20.24	3326007	79.62	3951943	94.61	319.53	1402
**SAMPLE_33**	5701898	1583567	27.77	4112106	72.12	5372858	94.23	390.83	1260
**SAMPLE_34**	4870290	1042338	21.40	3823458	78.51	4612145	94.70	370.58	1368
**SAMPLE_35**	4002138	751960	18.79	3245440	81.09	3770873	94.22	313.51	1296
**SAMPLE_36**	5393030	1416734	26.27	3966695	73.55	5096465	94.50	377.37	1240
**SAMPLE_37**	4041608	762968	18.88	3274249	81.01	3823807	94.61	312.94	1360
**SAMPLE_38**	5399666	1477613	27.36	3916460	72.53	5095500	94.37	367.92	1235

**SAMPLE_39**	3919572	875210	22.33	3042270	77.62	3691856	94.19	291.95	1293
**SAMPLE_40**	4332984	892022	20.59	3436977	79.32	4104886	94.74	334.20	1393
**SAMPLE_41**	3971380	781224	19.67	3186209	80.23	3775194	95.06	313.85	1380
**SAMPLE_42**	3732816	681963	18.27	3047544	81.64	3543952	94.94	295.02	1336
**SAMPLE_43**	3729244	686417	18.41	3040168	81.52	3516194	94.29	294.79	1391
**SAMPLE_44**	4124460	859458	20.84	3259705	79.03	3980052	96.50	331.44	1372
**SAMPLE_45**	5512724	1493879	27.10	4012552	72.79	5193536	94.21	375.35	1244
**SAMPLE_46**	4138582	966148	23.34	3165224	76.48	4009129	96.87	333.86	1405
**SAMPLE_47**	3538128	620712	17.54	2913541	82.35	3344114	94.52	281.04	1352
**SAMPLE_48**	4655008	1119190	24.04	3530653	75.85	4506732	96.81	370.56	1398
**SAMPLE_49**	4057396	851084	20.98	3202842	78.94	3912648	96.43	301.14	1317
**SAMPLE_50**	5203538	1297771	24.94	3899184	74.93	5037737	96.81	406.83	1322
**SAMPLE_51**	4869284	1268279	26.05	3595657	73.84	4708973	96.71	369.68	1425
**SAMPLE_52**	3748462	796364	21.25	2948236	78.65	3630872	96.86	315.76	1403
**SAMPLE_53**	4503968	1057702	23.48	3440000	76.38	4352802	96.64	355.35	1367
**SAMPLE_54**	1847982	294654	15.94	1550856	83.92	1786735	96.69	168.79	1343
**SAMPLE_55**	4747334	1181833	24.89	3558987	74.97	4594479	96.78	358.84	1285
**SAMPLE_56**	5811628	1595324	27.45	4208612	72.42	5626443	96.81	433.62	1419
**SAMPLE_57**	2693326	458770	17.03	2231811	82.86	2602974	96.65	237.62	1351
**SAMPLE_58**	3412974	692539	20.29	2715589	79.57	3293492	96.50	285.83	1283
**SAMPLE_59**	3538404	669174	18.91	2865978	81.00	3416385	96.55	279.16	1230
**SAMPLE_60**	5020500	1332814	26.55	3682305	73.35	4867239	96.95	380.88	1303
**SAMPLE_61**	4798092	1180693	24.61	3608026	75.20	4628447	96.46	359.22	1386
**SAMPLE_62**	4089686	880592	21.53	3204073	78.35	3945953	96.49	318.64	1340
**SAMPLE_63**	4095102	935844	22.85	3153206	77.00	3964662	96.81	334.22	1291
**SAMPLE_64**	5846254	1461675	25.00	4376723	74.86	5652672	96.69	428.66	1288
**SAMPLE_65**	5366774	1425054	26.55	3933705	73.30	5195364	96.81	409.48	1362
**SAMPLE_66**	2328898	341598	14.67	1984426	85.21	2251353	96.67	208.85	1296
**SAMPLE_67**	6109638	1701443	27.85	4401148	72.04	5903595	96.63	455.12	1316
**SAMPLE_68**	4722916	1144064	24.22	3573003	75.65	4558605	96.52	364.47	1358
**SAMPLE_69**	6218248	1779750	28.62	4432586	71.28	5866692	94.35	418.83	1207
**SAMPLE_70**	4310554	882439	20.47	3424267	79.44	4089256	94.87	335.73	1347
**SAMPLE_71**	3937946	753770	19.14	3180169	80.76	3730536	94.73	312.18	1346
**SAMPLE_72**	4986154	1284257	25.76	3698744	74.18	4707392	94.41	352.87	1314
**SAMPLE_73**	4061440	777417	19.14	3279642	80.75	3841684	94.59	320.33	1419
**SAMPLE_74**	4405010	1063782	24.15	3337005	75.75	4162780	94.50	319.51	1263
**SAMPLE_75**	5071674	1341029	26.44	3725592	73.46	4766355	93.98	352.68	1234
**SAMPLE_76**	4143456	753477	18.18	3384536	81.68	3976468	95.97	309.01	1346
**SAMPLE_77**	5138396	1201933	23.39	3930057	76.48	4953834	96.41	377.95	1279
**SAMPLE_78**	4782692	1027324	21.48	3749082	78.39	4611111	96.41	360.89	1331
**SAMPLE_79**	4838792	946789	19.57	3886423	80.32	4631115	95.71	343.38	1354

**SAMPLE_80**	4014524	805358	20.06	3204160	79.81	3868949	96.37	317.23	1354
**SAMPLE_81**	5525118	1478170	26.75	4040892	73.14	5349764	96.83	417.73	1320
**SAMPLE_82**	3579440	644938	18.02	2930753	81.88	3376713	94.34	288.06	1412
**SAMPLE_83**	3252646	533731	16.41	2716705	83.52	3070473	94.40	258.97	1387
**SAMPLE_84**	5332234	1431476	26.85	3895773	73.06	5043888	94.59	371.97	1310

The columns contain the following information: (1) SampleID, (2) total count of the sequenced reads, (3) number of reads marked as duplicates, (4) duplicates as a proportion of the total number of sequenced reads, (5) number of mapped reads without duplicates, (6) proportion of non-duplicated reads in the total number of sequenced reads, (7) number of reads mappinng to the reference genome with MAPQ at least 40, (8) proportion of the MAPQ40 and above reads in the total number of sequenced reads, (9) mean depth of coverage in the targeted regions, (10) number of called small variants.
